# Amelioration of human peritoneal mesothelial cell co-culture-evoked malignant potential of ovarian cancer cells by acacetin involves LPA release-activated RAGE-PI3K/AKT signaling

**DOI:** 10.1186/s11658-021-00296-3

**Published:** 2021-12-09

**Authors:** Meng Tian, Yingjie Tang, Ting Huang, Yang Liu, Yingzheng Pan

**Affiliations:** 1 Critical Care Medicine, Qingpu Branch of Zhongshan Hospital Affiliated to Fudan University, Shanghai, 201700 People’s Republic of China; 2 Department of Obstetrics, Chongqing Health Center for Women and Children, Chongqing, 401147 People’s Republic of China; 3 Department of Obstetrics and Gynecology, Second Affiliated Hospital, Chongqing Medical University, Chongqing, 400010 People’s Republic of China; 4 Department of Gynecological Endocrinology, Chongqing Health Center for Women and Children, No 120 Longshan Road, Yubei District, Chongqing, 401147 People’s Republic of China

**Keywords:** Ovarian cancer, Peritoneal mesothelial cells, Acacetin, PI3K/AKT, Cell invasion, Cell proliferation

## Abstract

**Background:**

Ovarian cancer is a devastating gynecological malignancy and frequently presents as an advanced carcinoma with disseminated peritoneum metastasis. Acacetin exerts anti-cancerous effects in several carcinomas. Here, we sought to investigate acacetin function in ovarian cancer malignancy triggered by peritoneal mesothelial cells.

**Methods:**

Peritoneal mesothelial cells were treated with acacetin, and then the conditioned medium was collected to treat ovarian cancer cells. Then, cell proliferation was analyzed by MTT assay. Transwell analysis was conducted to evaluate cell invasion. Protein expression was determined by western blotting. ELISA and qRT-PCR were applied to analyze inflammatory cytokine levels. The underlying mechanism was also explored.

**Results:**

Acacetin suppressed cell proliferation and invasion, but enhanced cell apoptosis. Furthermore, mesothelial cell-evoked malignant characteristics were inhibited when mesothelial cells were pre-treated with acacetin via restraining cell proliferation and invasion, concomitant with decreases in proliferation-related PCNA, MMP-2 and MMP-9 levels. Simultaneously, acacetin reduced mesothelial cell-induced transcripts and production of pro-inflammatory cytokine IL-6 and IL-8 in ovarian cancer cells. Mechanically, acacetin decreased lysophosphatidic acid (LPA) release from mesothelial cells, and subsequent activation of receptor for advanced glycation end-products (RAGE)-PI3K/AKT signaling in ovarian cancer cells. Notably, exogenous LPA restored the above pathway, and offset the efficacy of acacetin against mesothelial cell-evoked malignancy in ovarian cancer cells, including cell proliferation, invasion and inflammatory cytokine production.

**Conclusions:**

Acacetin may not only engender direct inhibition of ovarian cancer cell malignancy, but also antagonize mesothelial cell-evoked malignancy by blocking LPA release-activated RAGE-PI3K/AKT signaling. Thus, these findings provide supporting evidence for a promising therapeutic agent against ovarian cancer.

**Graphical Abstract:**

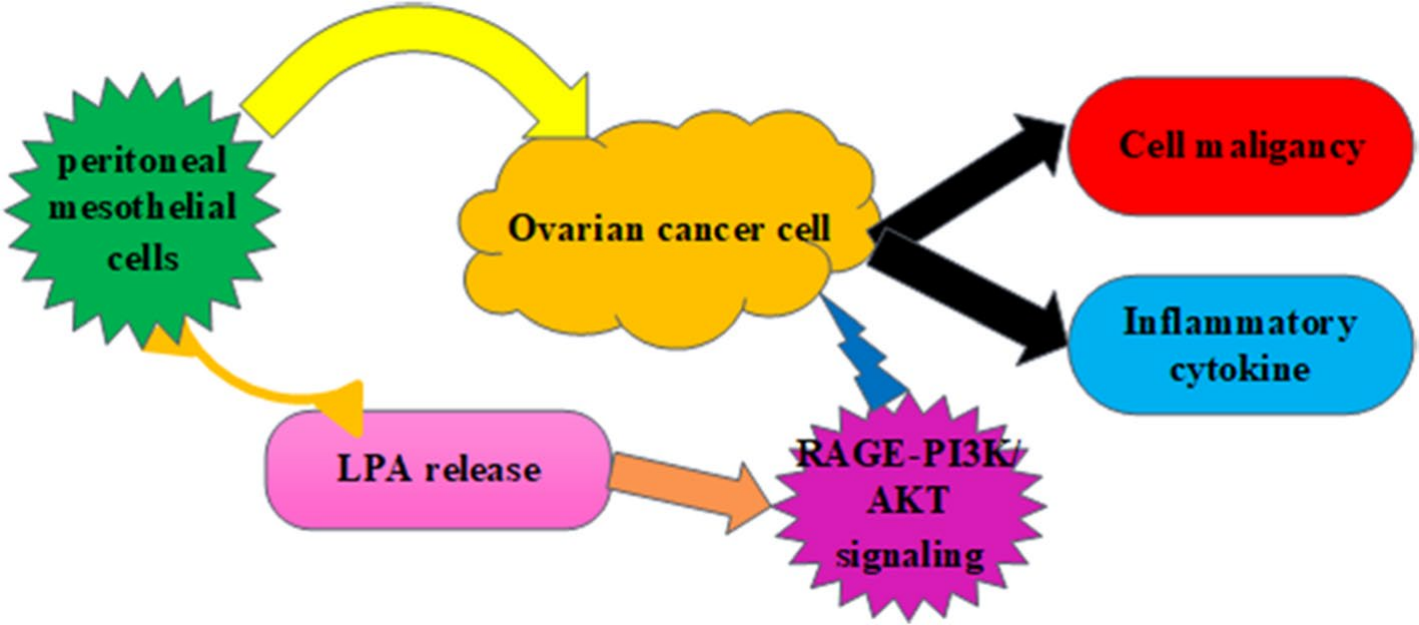

## Background

Ovarian cancer is the most lethal gynecological malignancy in the female reproductive tract, and is known as the fifth deadliest cancer worldwide [1]. Notably, epidemiologic research corroborates approximately 22,240 new diagnosed cases and 14,070 ovarian cancer deaths in the United States [2]. There is a steadily increasing incidence of ovarian cancer in the UK today, especially in women aged 65 and over [3]. Currently, high incidence and mortality of ovarian cancer constitute a proverbial obstacle for global health [1, 2]. Though advances in conventional therapy for ovarian cancer comprised surgery, radiotherapy and chemotherapy, more than 60% of patients are diagnosed with advanced disease [1]. Approximately 50–85% of patients with advanced ovarian cancer have a poor prognosis and experience recurrence within 5 years due to high metastasis characteristics, leading to an approximate median survival time of 2 years [1, 2].

It is a fact that high mortality of ovarian cancer often mainly results from the occult progression in the peritoneal cavity due to the preferential metastasis to the peritoneal cavity that constitutes a widely known condition named peritoneal ovarian carcinomatosis [4, 5]. Metastasis to the peritoneum is a critical step for the progression of ovarian cancer, based on the fact that it provides a nutrient-rich tumor microenvironment (TME) consisting of various cell types, such as fibroblasts and mesothelial cells [6, 7]. Initial research regarding TME usually focused on fibroblasts  [7]. Recently, increasing evidence has confirmed a critical contributor of peritoneal mesothelial cells to the development of ovarian cancer[6, 8]. Mesothelial cells rank as the major cell population within the peritoneum covering the superficial area [9, 10]. Emerging evidence has suggested that mesothelial cells can facilitate the progression of ovarian cancer by promoting multiple tumorigenic processes, including cell proliferation, invasion, migration and adhesion [8, 10, 11]. Therefore, there is an urgent need to elucidate the interplay and underlying mechanism between peritoneal mesothelial cells and ovarian cancer cells for cancer prevention.

**Fig. 1:**
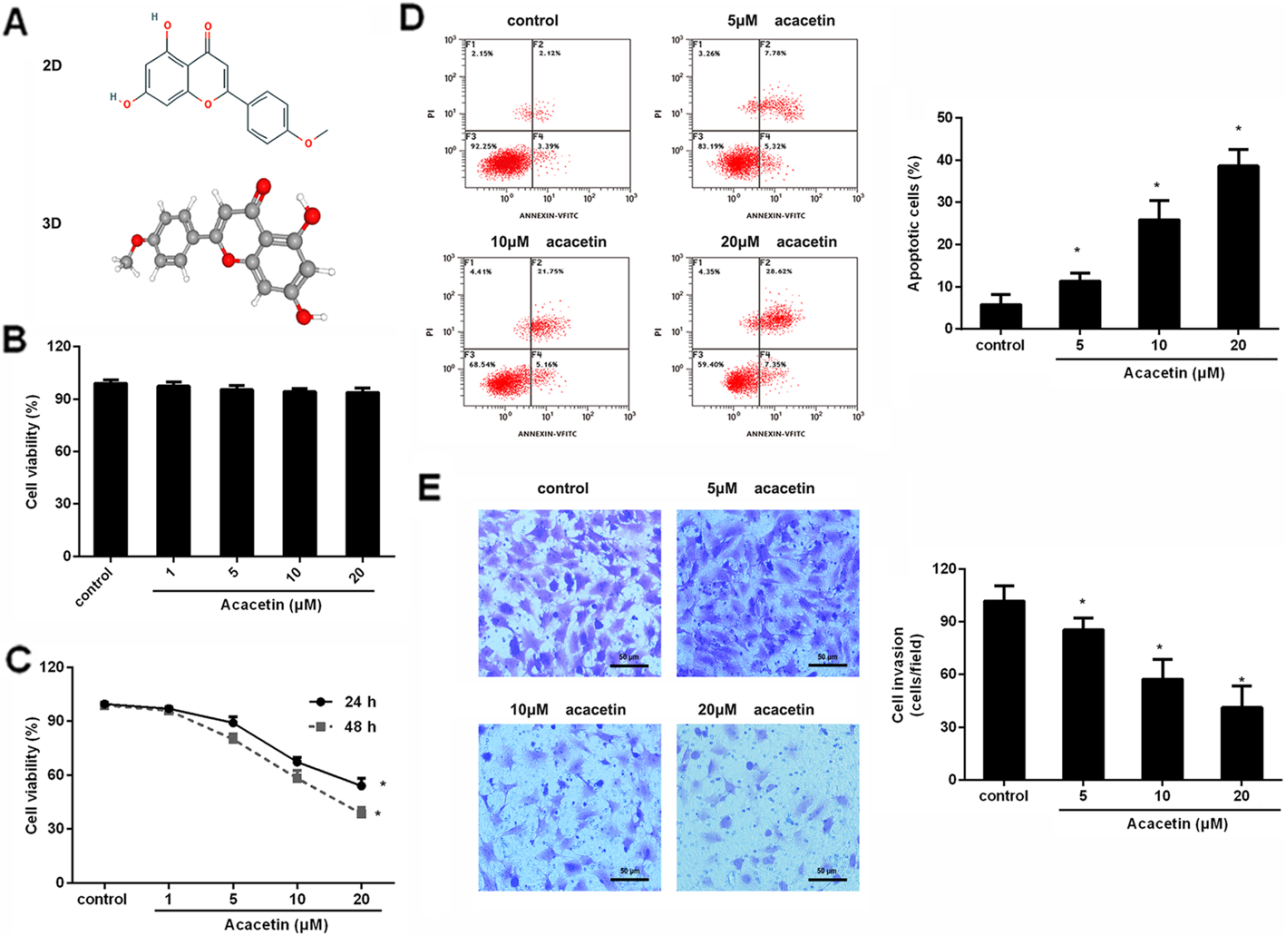
Acacetin restrained ovarian cancer cell malignancy. **A** Chemical structure of acacetin. **B** Normal human ovarian surface epithelial cell line IOSE80 was treated with the indicated doses of acacetin for 24 h; cell viability was then analyzed. **C** Human SKOV3 ovarian cancer cells were treated with various doses of acacetin (1–20 µM) for 24 and 48 h. Then, cell viability was determined by MTT assay. **D** After treatment with the indicated dose for 48 h, annexin V-FITC/PI staining was carried out to evaluate cell apoptosis. **E** Cell invasion was assessed by Transwell assay. *P < 0.05 vs. control group

An increasing body of attention has focused on the potential application of natural products that act as promising cancer therapeutic agents [12, 13]. Acacetin (5,7-dihydroxy-4ʹ-methoxyflavone) (Fig. 1A) is a common flavonoid compound that widely exists in plants, vegetables, seeds and flowers. Notably, previous findings have demonstrated that acacetin possesses anti-ischemia/reperfusion injury, anti-inflammatory and antioxidative activity [14, 15]. Recently, increasing evidence has indicated that acacetin exhibits anti-cancerous efficacy in several cancers, including skin cancer [16], breast cancer [17] and prostate cancer [13]. In particular, administration with acacetin restrains tumor angiogenesis and growth in ovarian cancer [18]. Nevertheless, little research is focused on its roles in the tumor microenvironment.

In the present study, we sought to investigate the efficacy of acacetin in peritoneal mesothelial cell-facilitated malignant potential in ovarian cancer cells. Additionally, the potential molecular mechanism was also elucidated.

## Methods

### Cell culture

The normal human ovarian surface epithelial cell line IOSE80, the human mesothelium cell line Met-5 A and the ovarian cancer cell line SKOV3 were bought from the American Type Culture Collection (ATCC; Manassas, VA, USA). For culture, the SKOV3 cells were maintained in RPMI-1640 medium containing 10% fetal bovine serum (FBS) (Thermo Fisher Scientific, Waltham, MA, USA) and 50 U/ml penicillin/streptomycin. The Met-5 A cells were grown in Dulbecco’s modified Eagle medium (DMEM)/F12 medium supplemented with 10% FBS, hydrocortisone (0.1 µg/ml), 50 U/ml penicillin/streptomycin, insulin (2.5 µg/ml) and 5 ng/ml EGF. All cells were housed in a humidified 5% CO_2_ atmosphere at 37 °C.

### Preparation of conditioned medium (CM) from mesothelium cells

Mesothelium cell line Met-5 A (1 × 10^5^ cells /ml) was grown to 60–70% confluence, and then was incubated with acacetin (C_16_H_12_O_5_; ≥ 97% purity; Sigma, St. Louis, MO, USA) (1, 5, 10 and 20 µM) for 24 h in the presence or absence of exogenous LPA (5 µM and 10 µM) [19] (Sigma). Subsequently, the conditioned medium was collected and filtered through a 0.22 μm filter to discard any cellular debris. Then, the medium was stored at -80ºC until required for subsequent experiments.

## Ovarian cancer cell treatment

Ovarian cancer SKOV3 cells were treated with acacetin (Sigma, St. Louis, MO, USA) at a range of concentrations varying from 1 µM to 20 µM and/or conditioned medium prepared from Met-5 A cells for 24 h.

### Cell proliferation analysis by MTT

SKOV3 ovarian cancer cells (2.5 × 10^4^ cells/well) were seeded into 96-well plates. Then, cells were treated with various doses of acacetin and conditioned medium from mesothelium cells. Approximately 24 h later, cells were cultured in RPMI-1640 medium supplemented with MTT reagent (5 mg/ml, Sigma) for 4 h. Afterwards, the formatted formazan precipitate was completely solubilized after addition of 100 µl of DMSO to each well for 15 min. Subsequently, a microplate reader (Bio-Rad, Hercules, CA, USA) was applied to capture the absorbance at 570 nm to evaluate cell viability.

### Assay of cell apoptosis by flow cytometer

After being seeded in 6-well plates, ovarian cancer cells were incubated with 5 µM, 1 µM and 20 µM of acacetin for 24 h. Then, cell apoptosis was assessed by an annexin V-FITC apoptosis analysis kit (Beyotime, Nantong, China). Briefly, the collected cells were centrifuged and re-suspended in 195 µl of binding buffer. Then, cells were incubated with 5 µl of annexin V-FITC and 10 µl PI at room temperature, avoiding light. Approximately 20 min later, a flow cytometer (BD Biosciences, CA) was introduced to discriminate cell apoptosis.

### Evaluation of cell invasion

To analyze cell invasion, a Matrigel-coated transwell chambers with 8 μm pore-size polycarbonate filters (BD Biosciences, Bedford, MA, USA) was applied. In brief, cells under acacetin, LPA and conditioned medium were collected and re-suspended in serum-free medium. After that, cells (1 × 10^5^ cells) were added to the upper chamber with Matrigel (1.5 mg/ml)-precoated transwell inserts, and invasion was allowed to occur. The lower chamber was supplemented with medium containing 10% FBS. Then, non-invading cells were removed from the upper chamber with a cotton swab. The invading cells were then fixed and stained with 0.1% crystal violet, and counted using a light microscope (× 200) in five fields/filter. All experiments were performed independently in triplicate.

### Immunoblotting

Cells that were treated with the indicated conditions were collected and lysed with RIPA lysis buffer. After centrifugation at 4 °C for 10 min, the extracted protein concentration was quantified using a BCA kit (Beyotime). Subsequently, 30 µg of protein was resolved by SDS-PAGE and subsequently transferred to a PVDF membrane (Millipore, Billerica, MA, USA). To prevent non-specific binding, the membrane was incubated with 5% non-fat milk. Then, the primary antibodies against PCNA, MMP-2, MMP-9, receptor for advanced glycation end-products (RAGE), p-AKT, AKT, p-PI3K and PI3K (all from Abcam, Cambridge, MA, USA) were added for further incubation at 4 °C overnight. Following rinsing with TBST three times, the membrane was treated with goat anti-rabbit secondary antibodies conjugated to horseradish peroxidase at room temperature for 2 h. The binding signal was visualized when exposed to chemiluminescence reagent (ECL, Beyotime). For normalization, β-actin was applied as an internal standard. The intensities of bands were then quantified using Image J software.

### RNA extraction and qRT-PCR analysis

After collection from various groups, total RNA from cells was prepared using the TRIzol reagent (Sigma). Then, the extracted total RNA was primed with oligo (dt) and reverse transcribed to synthesize the first-strand cDNA using a commercial SuperScript II First Strand Synthesis System Kit (Invitrogen, CA, USA). Afterwards, transcriptional levels of IL-6 and IL-8 were analyzed by real-time PCR on an Applied Biosystems 7300 Real-Time PCR System (Applied Biosystems; Foster City, CA, USA). All protocols were carried out according to instructions provided by a SYBR Premix Ex Taq II Kit (TaKaRa, Dalian, China). The specific primers for these genes were as follows: IL-6 (sense, 5ʹ-GACCACACTTGGAGGTTTAAGG-3ʹ; anti-sense, 5ʹ- CCACTGATCTGGTGGTGTAAAG-3ʹ), IL-8 (sense, 5ʹ-TTCACTGCTCTGTCGTACTTTC-3ʹ; anti-sense, 5ʹ-CACACCAAGGAAGGGTTCTTAT-3ʹ), and β-actin (sense, 5ʹ-TCCCTGGAGAAGAGCTATGA-3ʹ; anti-sense, 5ʹ-CAGGAAGGAAGGCTGGAAA-3ʹ). β-actin was introduced as an endogenous control to calculate target genes using the 2^−ΔΔCT^ method.

### ELISA assay

The supernatants from mesothelium cells and ovarian cancer cells were prepared after sonicating and centrifuged at 4 °C. Then, the contents of LPA in supernatants were measured using a Human lysophosphatidic acid (LPA) ELISA kit (Cusabio, Wuhan, China). Commercially available IL-6 and IL-8 ELISA kits (Invitrogen) were used to determine the levels of IL-6 and IL-8 in supernatants from ovarian cancer cells. All procedures were conducted according to the instructions of manufacturers.

### Statistical analysis

Results from at least three independent experiments are shown as mean ± SD. Statistical comparisons were performed by SPSS19.0, and determined using Student’s *t*-test for two groups and ANOVA with the post-hoc Student-Newman-Keuls test for three or more groups. The criterion for statistical significance was defined as P <0.05.

## Results

### Acacetin restrains ovarian cancer cell growth and invasion

To elucidate the function of acacetin in the microenvironment of ovarian cancer, we first evaluated cytotoxicity of acacetin on the normal ovarian surface epithelial cell line IOSE80 and found that acacetin had little cytotoxicity to IOSE80 cells with increasing doses (Fig. 1B). As presented in Fig. 1C, acacetin dose-dependently inhibited ovarian cancer cell viability with IC_50_ at 21.63 µM at 24 h and 13.65 µM at 48 h. Furthermore, exposure to acacetin dose-dependently suppressed ovarian cancer cell apoptosis relative to the control group (Fig. 1D). Transwell assay corroborated that cells treated with 5–20 µM acacetin restrained the number of invading cells in SKOV3 cells (Fig. 1E). Thus, these data indicate that acacetin may suppress the malignant progression of ovarian cancer.

### Acacetin incubation antagonizes mesothelial cell conditioned medium-induced pro-growth and invasion potential in ovarian cancer cells

**Fig. 2:**
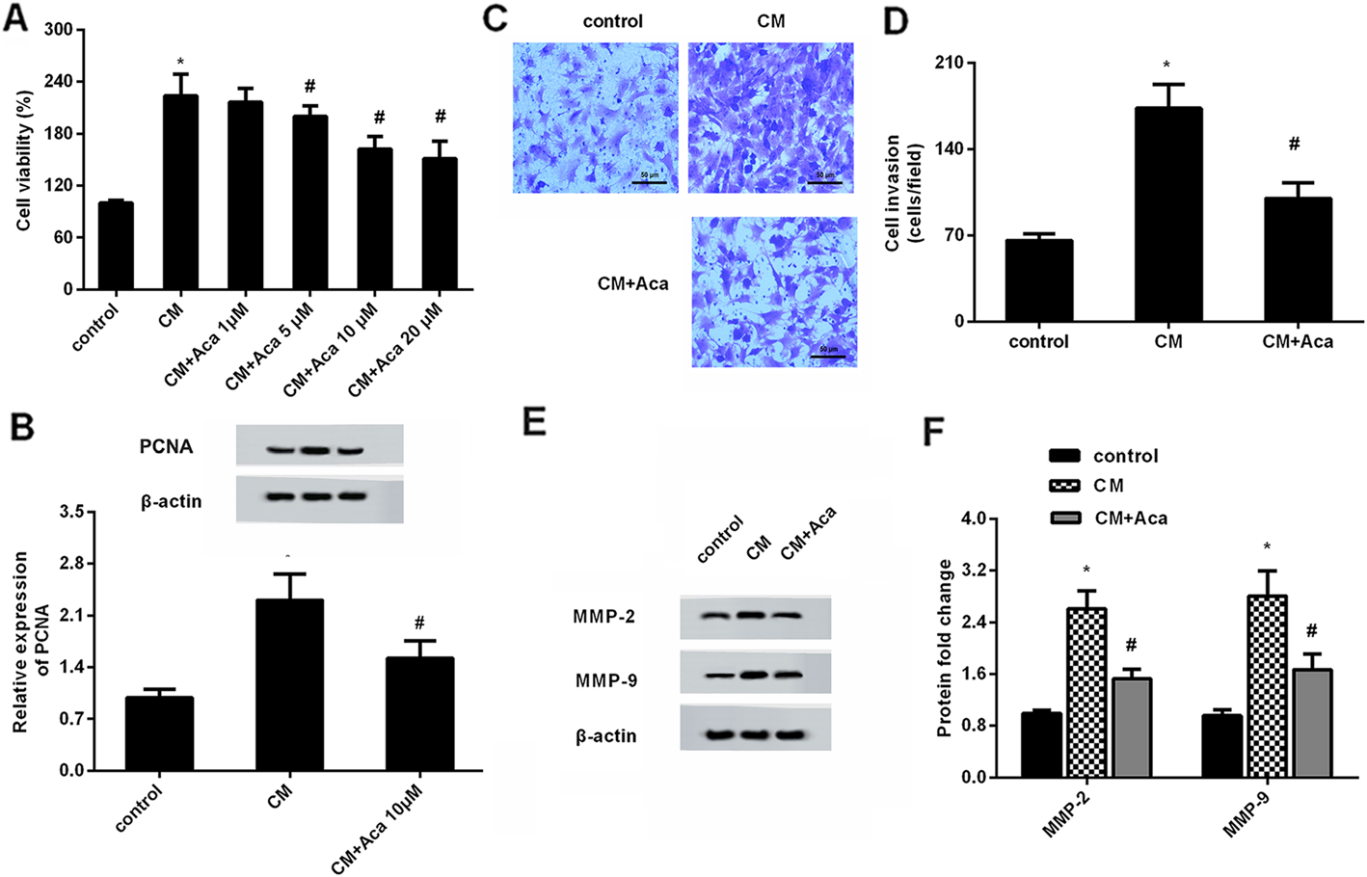
Administration with acacetin offset conditioned medium-induced ovarian cancer cell growth and invasion. **A** Ovarian cancer cells were incubated with conditioned medium (CM) from peritoneal mesothelial cells in presence of the indicated doses of acacetin. Approximately 48 h later, cell viability was detected by MTT assay. **B** The protein expression of proliferation-related PCNA was analyzed by western blotting. **C**, **D** Transwell assay was performed to evaluate cell invasion. **E**, **F** The corresponding effects on protein expression of MMP-2 and MMP-9. *P < 0.05

Convincing evidence substantiates carcinogenesis of mesothelial cells in the progression of ovarian cancer [8, 20]. We therefore next investigated the effect of acacetin on mesothelial cell-evoked malignant potential in ovarian cancer cells. As shown in Fig. 2A, conditioned medium (CM) from mesothelial cells increased cell viability approximately 223.6%-fold relative to the control group. Intriguingly, this up-regulation was abrogated when cells were incubated with CM from acacetin-treated mesothelial cells. Notably, there was no obvious difference between CM/10 µM acacetin and CM/20 µM acacetin groups. Concomitantly, CM exposure elevated the protein levels of proliferation-related PCNA in contrast to the control group, which was overturned after incubation with CM from 10 µM acacetin-treated mesothelial cells (Fig. 2B). Moreover, acacetin stimulation attenuated CM-induced ovarian cancer cell invasion (Fig. 2C, D), concomitant with the decrease in the protein levels of MMP-2 and MMP-9 (Fig. 2E, F).

### Treatment with acacetin offsets the transcription and release of inflammatory cytokine in response to CM from peritoneal mesothelial cells

**Fig. 3:**
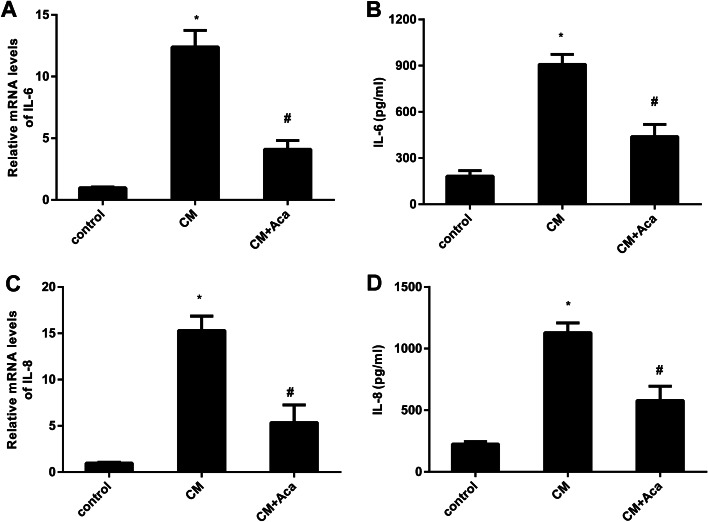
Acacetin antagonized mesothelial cell-evoked inflammatory cytokine production. **A** Ovarian cancer cells were treated with conditioned medium from mesothelial cells under acacetin condition (10 µM). Then, the mRNA levels of IL-6 were analyzed in ovarian cancer cells by qRT-PCR. **B** The production of IL-6 in ovarian cancer cells was quantified by ELISA assay. **C**, **D** The subsequent effects on IL-8 mRNA and release were measured. *P < 0.05 vs. control group, ^#^*P* < 0.05 vs. CM-treated group

As presented in Fig. 3A, ovarian cancer cells that were cultured in CM from peritoneal mesothelial cells exhibited increased mRNA levels in pro-inflammatory cytokine IL-6, which was offset after acacetin treatment. Simultaneously, acacetin-treated CM mitigated the production of IL-6 in ovarian cancer cells under CM conditions (Fig. 3B). Additionally, incubation with CM also enhanced the transcription (Fig. 3C) and release (Fig. 3D) of IL-8 in ovarian cancer cells. However, these increases were both weakened when cells were incubated with CM from acacetin-treated mesothelial cells.

### Administration with acacetin suppresses LPA releases from human peritoneal mesothelial cells

**Fig. 4:**
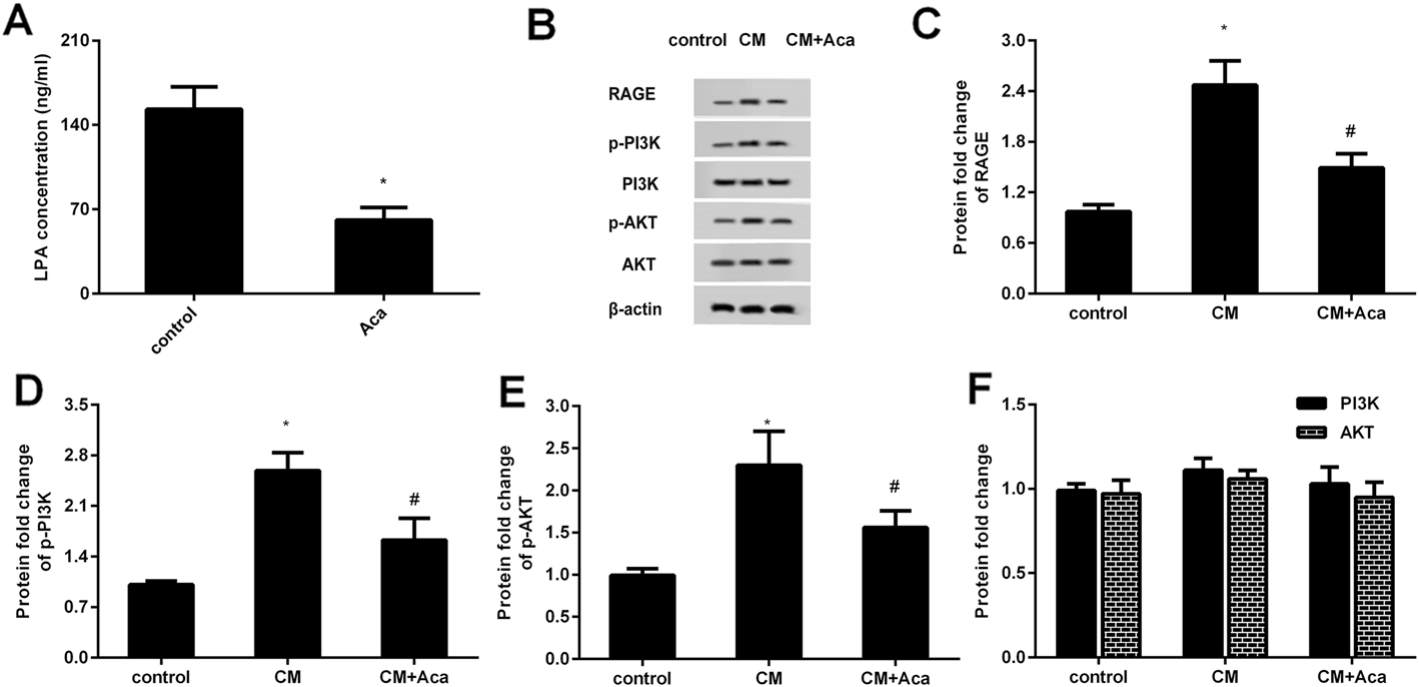
Acacetin inhibits LPA release from mesothelial cells and subsequent activation of RAGE-PI3K/AKT signaling. **A** The contents of LPA in conditioned medium (CM) from mesothelial cells were quantified. **B** After incubation with CM under acacetin condition, the protein expression of RAGE, p-PI3K, p-AKT was analyzed. **C**–**F** The corresponding quantified analysis of binding bands. *P < 0.05 vs. control group, ^#^*P* < 0.05 vs. CM-treated group

Accumulation evidence supports the critical roles of LPA in peritoneal mesothelial cell-mediated malignant progression of ovarian cancer cells [10, 20]. Therefore, we explored the effects of acacetin in LPA production from peritoneal mesothelial cells. It was observed that the acacetin treatment obviously inhibited LPA production in conditioned medium collected from peritoneal mesothelial cells (Fig. 4A).

### Acacetin inhibits CM-induced activation of RAGE-PI3K/AKT signaling in ovarian cancer cells

A previous study confirmed the crucial function of LPA in tumor growth in ovarian cancer by RAGE signaling. Thus, we further investigated the involvement of RAGE signaling during these processes. Importantly, CM incubation enhanced the protein expression of RAGE (Fig. 4B, C), as well as the down-stream p-PI3K (Fig. 4B, D) and p-AKT expression (Fig. 4B, E) in ovarian cancer cells, whereas no significant differences in PI3K and AKT protein levels were confirmed when cancer cells were incubated with CM (Fig. 4F). These findings suggest the inhibitory effects of acacetin on CM-activated RAGE-PI3K/AKT signaling.

### Exogenous supplementation of LPA overturns the effects of acacetin on mesothelial cell-evoked malignant potential in ovarian cancer cells

**Fig. 5:**
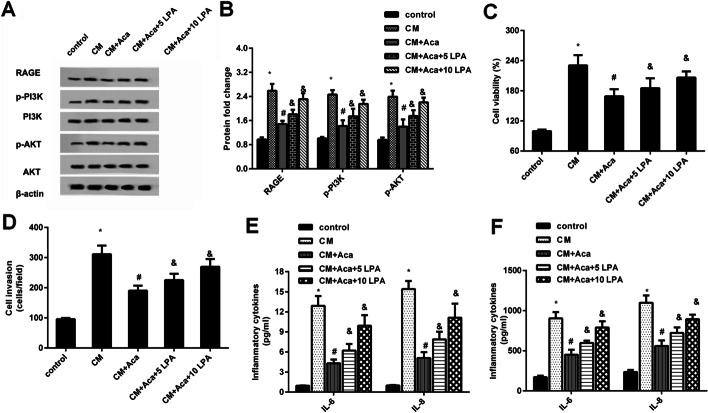
The LPA release-activated RAGE-PI3K/AKT signaling was responsible for mesothelial cell-mediated malignancy in ovarian cancer cells. **A**, **B** Following treatment with exogenous LPA, the activation of RAGE-PI3K/AKT signaling was determined in ovarian cancer cells under CM and acacetin conditions. Quantification of the above bands was analyzed by Image J software. **C**–**F** The subsequent effects on cell proliferation (**C**), invasion (**D**), the transcripts (**E**) and production (**F**) of IL-6 and IL-8were further determined. *P < 0.05 vs. control group, ^#^*P* < 0.05 vs. CM-treated group, ^&^*P* < 0.05 vs. CM-Aca group

To further decipher the involvement of LPA in acacetin function against mesothelial cell-evoked malignant potential in ovarian cancer cells, exogenous LPA was applied. Intriguingly, acacetin pretreatment restrained the expression of RAGE, p-PI3K and p-AKT protein in CM-stimulated ovarian cancer cells, which was reversed after supplementation with exogenous LPA at 5 µM and 10 µM (Fig. 5A, B). Moreover, the inhibitory efficacy of acacetin in CM-induced cell proliferation (Fig. 5C) and invasion (Fig. 5D) was overturned when cancer cells were incubated with CM containing added LPA. Simultaneously, LPA supplementation in CM abrogated acacetin-mediated suppression in CM-evoked elevation of mRNA levels (Fig. 5E) and release (Fig. 5F) of pro-inflammatory cytokines IL-6 and IL-8.

## Discussion

Currently, increasing insights have highlighted the promising therapeutic potential of natural products in cancer treatment, such as flavonoids [12]. Acacetin is a common natural plant-derived flavonoid compound, and exerts multiple medicinal benefits effects, including anti-oxidant, anti-neuronal injury and anti-inflammation [14, 15]. Intriguingly, accumulating evidence supports the anti-cancerous effects. For instance, acacetin treatment suppressed breast cancer cell growth [17]. In the current study, our findings revealed that acacetin suppressed ovarian cancer cell proliferation and invasion, but enhanced cell apoptosis. Intriguingly, a previous study confirmed the inhibitory effects of acacetin on angiogenesis and tumor growth in ovarian cancer [18]. Therefore, these data indicate that acacetin may act as a potential therapeutic agent for ovarian cancer prevention.

Ovarian cancer frequently presents as an advanced carcinoma with disseminated intra-abdominal metastasis, which is the major factor for patients to have a poor prognosis [4, 6]. Ovarian cancer predominantly undergoes a transcoelomic metastasis, where the primary tumor spreads throughout the peritoneal cavity [21]. Peritoneum metastasis marks a key step for tumor development because that peritoneum supports a nutrient-rich microenvironment for shed ovarian cancer cells [6]. Mesothelial cells are the major constituents of peritoneum covering the surface of the peritoneal cavity, and rank as the most abundant cell type in ascites of patients [22]. Recently, emerging evidence has confirmed the pro-tumor characteristics of mesothelial cells in the progression of ovarian cancer by enhancing cancer cell proliferation, invasion and adhesion [5, 10, 23]. Furthermore, mesothelial cells facilitate intraperitoneal invasiveness of ovarian malignancy and promote early ovarian cancer metastasis [8, 23]. Therefore, mediating the function of mesothelial cells in the microenvironment has become a new subject in ovarian cancer treatment. Analogously with previous research [10, 23], our findings corroborated the pro-proliferation and invasion potential of mesothelial cells in ovarian cancer. Intriguingly, acacetin antagonized mesothelial cell-evoked proliferation and invasion in ovarian cancer cells, concomitant with the decrease in MMP-2 and MMP-9 expression. Thus, acacetin may attenuate mesothelial cell-induced malignant potential in ovarian cancer cells.

Accumulating evidence has substantiated the involvement of the inflammatory response in the progression of cancer, including ovarian cancer [24]. Production of pro-inflammatory cytokine in ascites contributes to a more aggressive tumor phenotype [24, 25]. Notably, the present study confirmed that mesothelial cells enhanced the transcription and release of pro-inflammatory cytokines IL-6 and IL-8 in ovarian cancer cells. Recent findings demonstrated that high levels of IL-6 and IL-8 in peritoneal fluid are related to poor prognosis in ovarian cancer patients, confirming them as new prognostic biomarkers in ovarian cancer [26]. Moreover, IL-6 and IL-8 treatment enhances proliferation and metastatic potential of ovarian cancer cells, and facilitates ovarian cancer aggressiveness [27, 28]. Of interest, acacetin overturned mesothelial cell-evoked production of these two inflammatory cytokines.

Intriguingly, we next confirmed the high levels of LPA in conditioned medium of mesothelial cells. Like cancer-associated fibroblasts, human peritoneal mesothelial cells also secrete factors to facilitate tumor progression [6]. LPA is known as an essential microenvironmental factor in ovarian cancer, and increases in malignant ascites of ovarian cancer patients [29, 30]. Notably, a recent study corroborated the constitutive release of LPA from human peritoneal mesothelial cells, which can promote ovarian cancer malignancy by enhancing cell proliferation, invasion, migration and adhesion [10]. Additionally, LPA exposure induces pro-inflammatory cytokine production in ovarian cancer cells [19, 20, 25]. Here, treatment with acacetin suppressed LPA release from mesothelial cells.

Next, LPA release in conditioned medium from peritoneal mesothelial cells activated the RAGE-PI3K/AKT signaling in ovarian cancer cells. Previous research confirmed the overexpression of RAGE in ovarian cancer, suggesting it to be a useful biomarker of ovarian cancer prognosis [31]. Intriguingly, RAGE has previously been identified as a new receptor for LPA in ovarian cancer growth and oncogenic characteristics in glioma cells [32]. Activation of PI3K/AKT signaling by RAGE is involved in the initiation, chemoresistance and metastasis of cancer, including ovarian cancer [33, 34]. Moreover, the PI3K/AKT pathway has been proved to be associated with ovarian cancer-mesothelial adhesion [35]. Importantly, restoring the RAGE-PI3K/AKT signaling by exogenous supplementation with LPA offset the effects of acacetin against mesothelial cell-evoked proliferation, invasion and inflammatory cytokine production in ovarian cancer cells. Therefore, these findings suggest that acacetin may attenuate mesothelial cell-evoked malignant potential in ovarian cancer cells. Nevertheless, LPA also plays critical roles in carcinogenesis by binding to its receptors [36]. Thus, a further study will be performed to investigate the involvement of LPA receptor in acacetin-mediated anti-tumor efficacy in ovarian cancer microenvironment.

In summary, the present findings revealed that acacetin suppressed ovarian cancer cell growth and invasion. Additionally, treatment with acacetin also antagonized peritoneal mesothelial cell-evoked malignant potential of ovarian cancer cells by inhibiting cell proliferation and invasion via preventing LPA release-induced activation of RAGE-PI3K/AKT signaling. Consequently, the current data highlight the critical role of acacetin in the tumor microenvironment of ovarian cancer, implying a new therapeutic approach of acacetin against ovarian cancer. However, we just investigated the effect of acacetin on peritoneal mesothelial cell-evoked malignant potential of ovarian cancer cells in vitro. Does acacetin exert the ideal efficacy in the tumor microenvironment in vivo? This question will be addressed in our future work.

## Data Availability

All data generated or analyzed during this study are included in this published article.

## Abbreviations

DDP: Cisplatin
CM: Conditioned medium
LPA: Lysophosphatidic acid
RAGE: Receptor for advanced glycation end-products
TME: Tumor microenvironment
FBS: Fetal bovine serum
